# Random Chromosome Partitioning in the Polyploid Bacterium *Thermus thermophilus* HB27

**DOI:** 10.1534/g3.119.400086

**Published:** 2019-02-21

**Authors:** Haijuan Li

**Affiliations:** College of Biological and Environmental Engineering, Xi’an University, Xi’an 710065, Shaanxi, China

**Keywords:** polyploid prokaryotes, *Thermus thermophilus*, random chromosome segregation, ParA and MreB homologs

## Abstract

Little is known about chromosome segregation in polyploid prokaryotes. In this study, whether stringent or variable chromosome segregation occurs in polyploid thermophilic bacterium *Thermus thermophilus* was analyzed. A stable heterozygous strain (HL01) containing two antibiotic resistance markers at one gene locus was generated. The inheritance of the two alleles in the progeny of the heterozygous strain was then followed. During incubation without selection pressure, the fraction of heterozygous cells decreased and that of homozygous cells increased, while the relative abundance of each allele in the whole population remained constant, suggesting chromosome segregation had experienced random event. Consistently, in comparison with *Bacillus subtilis* in which the sister chromosomes were segregated equally, the ratios of DNA content in two daughter cells of *T. thermophilus* had a broader distribution and a larger standard deviation, indicating that the DNA content in the two daughter cells was not always identical. Further, the protein homologs (*i.e.*, ParA and MreB) which have been suggested to be involved in bacterial chromosome partitioning did not actively participate in the chromosome segregation in *T. thermophilus*. Therefore, it seems that protein-based chromosome segregation machineries are less critical for the polyploid *T. thermophilus*, and chromosome segregation in this bacterium are not stringently controlled but tend to be variable, and random segregation can occur.

An increasing number of bacterial and archaeal species are recognized as being oligo- or polyploid, *e.g.*, containing on average more than one chromosomal copy per cell. Vastly different levels of the number of genome equivalents per cell have been measured in microorganisms belonging to such distinct groups as the *Archaea*, *Cyanobacteria* and *Proteobacteria* (Breuert *et al.* 2006; Schneider *et al.* 2007; Hildenbrand *et al.* 2011; Pecoraro *et al.* 2011). The reported genome copy numbers in these polyploid microorganisms range from two to several hundred, and there are extreme cases with several thousand copies, like in the giant endosymbiont bacterium *Epulopiscium* sp. (Mendell *et al.* 2008). It has been previously shown that a certain strain of *T. thermophilus*, a member of the deep-branching *Deinococcus-Thermus* group, which has been established as a model thermophilic organism, is also polyploid, carrying four to five genome copies per cell under slow growth condition, these genome copies are assumed to be genetically identical (Henne *et al.* 2004; Ohtani *et al.* 2010). Almost 40 years ago, the same ploidy level has been determined for the related radiation-resistant bacterium *Deinococcus radiodurans* (Hansen 1978). Several possible evolutionary advantages of having more than one genome copy have been suggested. For example, genome redundancy is associated with higher resistance against DNA damaging agents and permits the accumulation of otherwise deleterious recessive mutations (Comai 2005; Pecoraro *et al.* 2011; Soppa 2013, 2014). An important question is how polyploid microorganisms partition the multiple genome copies to daughter cells. The mechanism of genome partitioning has been extensively studied in model bacteria like *Escherichia coli* (Lampo *et al.* 2015; Cass *et al.* 2016), *Bacillus subtilis* (Kloosterman *et al.* 2016; Wang *et al.* 2017) and *Caulobacter crescentus* (Taylor *et al.* 2017), which are monoploid (only very slowly growing *E. coli* cells are truly monoploid). In a stark contrast, very few literatures could be found describing chromosomal dynamics in bacterial cells with more than two complete chromosome copies. Basically, chromosome partitioning in polyploid bacteria could be either stringent or variable (*e.g.*, random). There is an additional layer of complexity in a dividing polyploid cell – if, for some reason, the cell carries two different alleles at the same locus (heterozygous), there would be different genetic outcomes in the progeny depending on the basic mode of chromosome partitioning. In a stringent mode, each chromosomal copy is actively passed on to the recipient cells via various partitioning mechanisms. Such a partitioning mode would lead to progeny which is genetically identical to the parents. If the chromosomes are divided randomly in the daughter cells, similar to high-copy-number plasmids, these cells can receive any combination and/or different numbers of the parental genomes. An important consequence of this mode would be that, if the parental cell was heterozygous, the daughter cells can be genetically different from the parent due to allele segregation. Such a mode has been proposed for polyploid euryarchaeota *Methanococcus jannaschii* (Malandrin *et al.* 1999), and polyploid cyanobacteria *Anabaena* sp. 7120 (Hu *et al.* 2007) and *Synechocystis* sp. (Schneider *et al.* 2007) based on DNA content analysis in dividing cells.

In *T. thermophilus*, it is not known whether the chromosome copies segregate into daughter cells via a stringent or variable mode. In contrast to some polyploid cyanobacteria, the genomes of so far sequenced members of the *Thermales* order (including *T. thermophilus*) contain homologs of the actin-like MreB protein as well as presumably complete chromosomal ParAB - *parS* systems, which have been suggested to be involved in active genome partitioning in many bacterial species. For instance, in *E. coli* it has been shown that MreB is important for both chromosomal bulk DNA and origin segregation (Kruse *et al.* 2003, 2005, 2006; Madabhushi and Marians 2009). Similarly, depletion of *mreB* in *B. subtilis* and *C. crescentus* leads to considerable defects in chromosome segregation, where replication origins fail to localize correctly (Soufo and Graumann 2003; Gitai *et al.* 2004, 2005; Shebelut *et al.* 2009; Sundararajan and Goley 2017). Disruptions of *parAB* in the chromosomal ParAB - *parS* systems also cause defects in chromosome segregation in many bacteria, *e.g.*, in *B. subtilis* (Ireton *et al.* 1994; Sharpe and Errington 1996; Lewis and Errington 1997; Lee and Grossman 2006; Murray and Errington 2008; Sullivan *et al.* 2009; Gruber and Errington 2009; Scholefield *et al.* 2011), *C. crescentus* (Mohl and Gober 1997; Mohl *et al.* 2001; Toro *et al.* 2008; Ptacin *et al.* 2010; Shebelut *et al.* 2010), *Streptomyces coelicolor* (Kim *et al.* 2000; Jakimowicz *et al.* 2002), *Vibrio cholera* (Fogel and Waldor 2006; Yamaichi *et al.* 2007), *Pseudomonas aeruginosa* (Lasocki *et al.* 2007), *Corynebacterium glutamicum* (Donovan *et al.* 2010), *Streptococcus pneumonia* (Minnen *et al.* 2011) and *Mycobacterium smegmatis* (Ginda *et al.* 2013). In this manuscript, the mode of chromosome segregation in *T. thermophilus* HB27 was investigated. A heterozygous strain in which two antibiotic resistance alleles could be stably maintained in the presence of selection pressure was initially generated. The inheritance of the two alleles in the absence of selection was then followed in the daughter cells by molecular and genetic techniques. The results from these experiments suggested that in *T. thermophilus*, partitioning of the chromosomal copies would experience random event. Analysis of dividing nucleoids in *T. thermophilus* and *B. subtilis* by fluorescence microscopy further confirmed that the chromosome segregation occurred less stringently in *T. thermophilus* than in *B. subtilis*. In addition, through generating Δ*mreB*Δ*parA* double deletion mutant followed by phenotype analysis with respect to chromosome segregation, it was found that in *T. thermophilus* both the ParA and MreB homologs were not actively participated in chromosomal bulk DNA segregation. The data from these experiments strongly suggest that the protein-based chromosome segregation machineries are less important for the polyploid *T. thermophilus* cells, and the chromosome segregation in this bacterium is likely non-stringent or often irregular.

## Materials And Methods

### Bacterial strains and growth conditions

*E. coli* DH5α was used as a host strain for plasmid constructions and was grown at 37° in LB medium (contains 10 g/L tryptone, 5 g/L yeast extract, 5 g/L NaCl). *T*. *thermophilus* HB27 wild type (DSM 7039) and derivative strains were grown at 70° or 60° in TB medium. TB medium contained 8 g/L trypticase peptone (BD Biosciences, Heidelberg, Germany), 4 g/L yeast extract, and 3 g/L NaCl, and had a pH of 7.5. *B. subtilis* 168 (DSM 402) was grown in LB medium at 30°. The growth media were supplemented with ampicillin (100 µg/mL for *E. coli*), kanamycin (50 µg/mL for *E*. *coli* and 20 µg/mL for *T*. *thermophilus*), bleomycin (“Bleocin”, Calbiochem, 15 µg/mL for *E*. *coli* and 3 µg/mL for *T*. *thermophilus*) or X-Gluc (5-bromo-4-chloro-3-indolyl-β-D-glucopyranoside, 100 µg/mL) when necessary.

### Determination of chromosome copy numbers

The real-time quantitative PCR was essentially performed based on the method described (Breuert *et al.* 2006; Li *et al.* 2015). Specifically, two loci (TTC1610 (near *oriC*) and TTC0574 (closer to *terC*) on the chromosome were chosen as the investigation targets. Approximately 1 kbp standard fragments of the two loci were PCR amplified using *T. thermophilus* genomic DNA as a template. The PCR products were purified from agarose gels followed by photometrical measurement to determine the concentrations (Nanodrop 2000). The molecular concentrations of the standard fragments were calculated using the online software “oligo calc” (www.basic.northwestern.edu/biotools). A series of dilutions of the standard fragments containing defined numbers of molecules were used in the real time PCR analyses. Standard curves were then generated by plotting the C_t_ values against the corresponding molecular concentrations in the reaction (molecules/μL). Quantified cell numbers (determined both by spectrophotometry and Neubauer counting chamber) from exponentially growing cultures were harvested and lysed by cell lysis buffer, the cell lysis efficiency was determined by cell counting. The cell lysates were then dialyzed, and aliquots from the dilution series of the cell lysates were used as templates in the real time PCR reactions. For real time PCR reactions, fragments of 139 bp (TTC1610) and 119 bp (TTC0574) were amplified, and three independent repeats were performed. Eventually, the chromosomal copy numbers per cell were calculated based on the created standard curves and the cell density.

### Plasmid construction

All plasmids and strains used are listed in Table 1 and all primers used are listed in Table 2. For construction of pUC-Δ42, the flanking regions about 1 kbp of the *bgl* gene (TTP0042, encoding β-glucosidase) were respectively PCR amplified from *T. thermophilus* HB27, using primers that permitted to generate sufficient overlaps between each other; the two flanking regions were then cloned into *Xba*I digested pUC18 vector via Gibson Assembly method (New England Biolabs) (Gibson *et al.* 2009). The plasmids pCT3FK-2 and pJ-Δ*pyrE*::*blm* were used in the generation of the stable heterozygous strain HL01. pCT3FK (Angelov *et al.* 2009) in which a kanamycin resistance marker (*kat*) was sandwiched by two flanking regions of *pyrE* gene (TTC1380) was mutagenized (Change-IT Multiple Mutation Site Directed Mutagenesis Kit, Affymetrix, USA) to introduce two *Zra*I sites in the flanking sequences, followed by restriction and re-ligation to excise 521 bp of flanking sequences, while maintain the native *pyrE* flanking regions intact, this gave pCT3FK-2. For generation of pJ-Δ*pyrE*::*blm*, a 2.3 kbp *pyrFE* region was PCR amplified and cloned in pJET1.2 giving pJ-*pyrFE*; two *Nde*I sites were introduced to pJ-*pyrFE* by the same mutagenesis tool at positions -3 and +551 relative to the ORF of the *pyrE* gene. The mutagenized pJ-*pyrFE* was then digested with *Nde*I to get rid of the *pyrE* gene, and ligated with a bleomycin resistance marker *blm* (sequence data from Brouns *et al.* 2005). The plasmid pMK-*parB-sgfp* allowing expression of ParB-sGFP in *T. thermophilus* was constructed in a former study (Li *et al.* 2015). The plasmids pUC-Δ*mreB*::*kat* and pUC-Δ*parA*::*blm* were gene deletion vectors for generation of double knockout mutant Δ*mreB*::*kat*/Δ*parA*::*blm* in *T. thermophilus* HB27. The two flanking regions (about 1 kbp) of the *parA* (TTC1605) and *mreB* (TTC1464) genes, and the *blm*/*kat* cassette were respectively amplified by PCR, using *T. thermophilus* HB27 genomic DNA and plasmids containing the *blm*/*kat* cassettes (de Grado *et al.* 1999; Brouns *et al.* 2005) as the templates. Gibson assembly reaction was used to assemble the three PCR fragments into *Xba*I-digested pUC18 respectively, as they contained sufficient overlaps between each other.

**Table 1:** Strains and plasmids used in this study

Name	Description*^a^*	Reference
Plasmids		
pUC-Δ42	clean deletion vector for generating homozygous and heterozygous Δ*bgl* in *T. thermophilus* HB27, *ori* pUC, Amp^R^	this study
pCT3FK-2	allele exchange vector for generating Δ*pyrE*::*kat* and HL01, *ori* pBR322, Km^R^	this study
pJ-*pyrFE*	intermediate vector for generating pJ-Δ*pyrE*::*blm*, *ori* pJET, Amp^R^	this study
pJ-Δ*pyrE*::*blm*	allele exchange vector for generating Δ*pyrE*::*blm* and HL01, *ori* pJET, Amp^R^, Blm^R^	this study
pMK-*parB-sgfp*	pMK18 derived vector, allowing expression of parB-sGFP in *T. thermophilus*, *Tth* (*repA*), *Ec* (*oriE*), Km^R^	Li *et al.* 2015
pUC-Δ*parA*::*blm*	allele exchange vector for generating Δ*parA*::*blm* in *T. thermophilus* HB27, *ori* pUC, Amp^R^, Blm^R^	this study
pUC-Δ*mreB*::*kat*	allele exchange vector for generating Δ*mreB*::*kat* in *T. thermophilus* HB27, *ori* pUC, Amp^R^, Km^R^	this study
Strains		
HB27	*T. thermophilus* wild type	DSM 7039
HB27 wt/Δ-*bgl*	*T. thermophilus* HB27 heterozygous derivative with *bgl* clean deleted	this study
HB27 Δ*pyrE*::*kat*	*T. thermophilus* HB27 homozygous derivative with *pyrE* replaced by *kat*	this study
HB27 Δ*pyrE*::*blm*	*T. thermophilus* HB27 homozygous derivative with *pyrE* replaced by *blm*	this study
HL01	*T. thermophilus* HB27 heterozygous derivative with *pyrE* replaced by *kat* and *blm* simultaneously	this study
HB27/ParB-sGFP	*T. thermophilus* HB27 derivative permitting expression of ParB-sGFP	Li *et al.* 2015
Δ*mreB*::*kat*/Δ*parA*::*blm*	*T. thermophilus* HB27 derivative with *parA* replaced by *blm* and *mreB* replaced by *kat*	this study
168	*B. subtilis* wild-type strain	DSM 402

a *Ec* (*oriE*), replication origin for *E. coli*; *Tth* (*repA*), replication origin for *T. thermophilus*.

**Table 2:** Primers used in this study

Name	Sequences (5′-3′)*^a^*	Usage
1610-F	TCAAGGAGAAGGGCTACAG	generating standard fragment for qPCR in the TTC1610 region
1610-R	CCTTGTAGCTCACGGAAAC	
1610-F-1	ACGCCATCCTGGTCAAGGTG	primers of qPCR reactions for detecting TTC1610 copy numbers
1610-R-1	AGGTCGGCGATGAAGCTGTC	
0574-F	CCGGCAGGTAGACGTCAAAG	generating standard fragment for qPCR in the TTC0574 region
0574-R	TGAGCCGGAGGGAGTTTGAG	
0574-F-1	GTGACCACCACGCTTTCGGG	primers of qPCR reactions for detecting TTC0574 copy numbers
0574-R-1	TTAGGCCGCCAGGATCAGTACG	
bglF1-F	tgcatgcctgcaggtcgactAGACCATCCCCCAGGAGCTC	amplifying *bgl* upstream flanking region for pUC-Δ42
bglF1-R	cctctggcggggcacttagCTCGGTCATAGGCGTTTCTC	
bglF2-F	gagaaacgcctatgaccgagCTAAGTGCCCCGCCAGAGG	amplifying *bgl* downstream flanking region for pUC-Δ42
bglF2-R	gctcggtacccggggatcctGCCAGAACCAGAACGAAAAG	
dbgl-F	GCCGTCTACATCTTCCTCAC	PCR determination of *bgl* deletion
dbgl-R	TACCTTCCCGAGGACATCAC	
pCT3FKm-Zra-F	ACCTGCA**GACGTC**CAAGCTTGGCACTGGCCGTCGTTTTACAA	site-directed mutagenesis of pCT3FK
pCT3FKm-Zra-R	TTTCAGCA**GACGTC**GTTTCCTTTCTTTTCAGAGGGTATTTTA	
pyr.F	CCGAGCCCTTGGCCCATATC	cloning of the *pyrFE* region
pyr.R	CAGGACCGCCACCCTCATA	
pyrEm-Nde.F	GAGGAAGCG**CATATG**AGACCTCCTCC	site-directed mutagenesis of pJ-*pyrFE*
pyrEm-Nde.R	CAGGACGTC**CATATG**CCCCTACTCTAC	
pyrF.R	GGACCCTCCCGGTACCTTTC	combing with pyr.F used for probe generation for Southern blot
pyr.R2	GCTTTCCAGGTTGACGGTAAGC	combing with pyr.F used for PCR detection of *pyrE* gene replacements
mreB-1-F	catgcctgcaggtcgactAAACGGGACCGATTCCTC	amplifying *mreB* downstream flanking region for pUC-Δ*mreB*::*kat*
mreB-1-R	agagcgcccaatacgcaaaccGAGCTCGCCTCGGACATCTAC	
mreB-2-F	cttggaggagaaacgccTGCCGATGTCTTCGCCTTTAAGC	amplifying *mreB* upstream flanking region for pUC-Δ*mreB*::*kat*, and Southern blot probe template generation for *mreB* deletion detection
mreB-2-R	cggtacccggggatcctGGGTGGACCTCATCATTGAC	
kat-F	cggtttgcgtattgggcgctctTCCCCGGGAGTATAACAGAAACC	amplifying *kat* for pUC-Δ*mreB*::*kat*
kat-R	ggcgtttctcctccaagAATTCCGTTCAAAATGGTATG	
dmreB-F	TTGAGGATCTCCCGGATGTC	PCR determination of *mreB* deletion
dmreB-R	ATCGCGAGCCGCATTGAGAA	
parA-F1-F	tgcatgcctgcaggtcgactTCCTCCAAGGAGCGGTACTG	amplifying *parA* upstream flanking region for pUC-Δ*parA*::*blm*
parA-F1-R	gttatactcccggggatcccCCTGGCAGAGGAGGTGATGG	
parA-F2-F	gactgatctagaggatccccGCCCTTAGCATAACGGATAC	amplifying *parA* downstream flanking region for pUC-Δ*parA*::*blm*
parA-F2-R	cggtacccggggatcctCAAGTACGCGGGCTACATTG	
blm-F	GGGATCCCCGGGAGTATAAC	amplifying *blm* for pUC-Δ*parA*::*blm*
blm-R	GGGGATCCTCTAGATCAGTC	
probe-F	CCTCGGCTTCCTCAAGCTCTTC	Southern blot probe template generation for *parA* deletion detection
probe-R	CCGAAGAGGACGCGCACCGC	
dparA-F	TAGCGCCTTTCCCCCGCCAC	PCR determination of *parA* deletion
dparA-R	ACCTGGTGGTGTTGGAGAAG	

a Enzyme restriction sites are in bold and sequences that create the overlaps for the Gibson assembly reactions are in lowercase.

### **Determination of the genetic outcomes of the heterozygous** strain **wt/**Δ-bgl

The gene clean deletion vector pUC-Δ42 was linearized by *Hin*dIII and transformed into *T. thermophilus* HB27 based on the protocol described by de Grado *et al.* (1999). The transformation reaction was appropriately diluted and plated on TB agar plates supplemented with X-Gluc. After incubation at 70° for 2 d, colonies that exhibited intermediate color were selected for genotype analysis. The correct heterozygous strain (wt/Δ-*bgl*) was then grown in TB medium for 24 h and the culture was restreaked on TB-X-Gluc plates. The phenotype of the progeny was discriminated by colony color, and the genotype was determined by PCR.

### Generation of homozygous T. thermophilus gene deletion mutants

The allele exchange vectors pCT3FK-2 and pJ-Δ*pyrE*::*blm* were linearized by *Sca*I and *Hin*dIII respectively, then transformed into *T. thermophilus* HB27 in the same manner. The transformation reactions were respectively streaked on TB agar plates supplemented with kanamycin or bleomycin. Several colonies were randomly selected, and PCR (using primers flanking the *pyrE* gene) was used to sort out the colonies which only contained the mutant allele. This resulted in two homozygous strains, *i.e.*, Δ*pyrE*::*kat* and Δ*pyrE*::*blm*, which carried either the *kat* or the *blm* resistance marker at the *pyrE* gene locus. For generation of Δ*mreB*::*kat*/Δ*parA*::*blm* mutant, single Δ*mreB*::*kat* mutant was initially created. The pUC-Δ*mreB*::*kat* vector was linearized by *Hin*dIII and transformed into wild-type *T. thermophilus* cells followed by plating on agar plates containing kanamycin. Several transformants were exposed to PCR analysis to determine mutants containing homozygous Δ*mreB*::*kat* allele. Linearized pUC-Δ*parA*::*blm* was sequentially transformed into the correct Δ*mreB*::*kat* strain, transformants were selected on agar plates containing both antibiotics. The homozygous double deletion mutant Δ*mreB*::*kat*/Δ*parA*::*blm* was prescreened by PCR and further confirmed by Southern blot.

### Construction of a stable heterozygous strain and kinetics of allele segregation

The heterozygous strain HL01 was obtained by transforming linearized pJ-Δ*pyrE*::*blm* to the homozygous Δ*pyrE*::*kat* strain and selecting on TB agar plates supplemented with both kanamycin and bleomycin. The heterozygous strain was initially verified by PCR as well, and further confirmed by Southern blot and DNA sequencing of the respective genomic regions. The allele segregation experiment was performed by growing cells of HL01 in 5 mL of TB medium supplemented with both antibiotics for 12 h, followed by washing the cells three times in 1 × PBS buffer and inoculation in 50 mL antibiotic-free TB medium (time point 0). Growth was continued for 120 h at 70° with agitation. Samples (2 mL) were taken at various time points as indicated in the results to determine the fraction of each phenotype by spreading the cells on TB plates (without antibiotics) and re-streaking the colonies on plates supplemented with kanamycin or bleomycin. The same colony was parallelly streaked on TB plate to determine the growth. The relative amount of each allele of the samples was estimated by Southern blot followed by band intensity measurements (Image J, NIH, USA).

### Fluorescence microscopy

To measure the relative DNA content in two dividing cells by fluorescence microscopy, the *T*. *thermophilus* strains (HB27, HB27/ParB-sGFP, and HB27 Δ*mreB*::*kat*/Δ*parA*::*blm*) and *B. subtilis* 168 cells were grown to exponential phase. Cells from 0.5 mL cultures were washed once in 1 × PBS buffer, fixed by ethanol, and resuspended in the same volume of PBS buffer. When necessary, the DNA-specific dye DAPI (4′,6-diamidino-2-phenylindole dihydrochloride) and CFS (6-carboxyfluorescein) which stains the cell membrane were added to the cell suspension with final concentrations of 0.2 µg/mL and 10 µg/mL respectively, followed by incubating at room temperature for 30 min. The residual dyes were removed by washing the cells three times with 1 × PBS buffer. After mounting the cells on glass slides, fluorescence microscopy was carried out (Zeiss Axio Imager M1). The filter “DAPI” was used to observe DAPI-stained DNA signal, and the filter “AF488” was used to observe CFS-stained membrane signal and ParB-sGFP signal. The acquired micrographic images were analyzed with the AxioVision software (Carl Zeiss, Germany) and Image J (NIH, USA).

### Data availability

The author affirm that all plasmids are available upon request, and all data necessary for confirming the results of the article are included within the article, figures and tables. Supplemental material available at Figshare: https://doi.org/10.25387/g3.7746878.

## Results

### Real-time **PCR determination of the chromosomal copy number of T. thermophilus**

The *T*. *thermophilus* strain HB8 has been reported to contain four to five copies of chromosomes per cell under slow growth condition (Ohtani *et al.* 2010). For the *T*. *thermophilus* type strain HB27, the chromosomal copy numbers are not clearly reported. Using real-time PCR method, the chromosomal copy numbers of *T*. *thermophilus* HB27 during exponential growth in rich medium was determined. This real-time PCR method was developed by Breuert *et al.* (2006), and has been used to determine the exact genome copy numbers in the cell populations of various archaeal and bacterial species (Hildenbrand *et al.* 2011; Pecoraro *et al.* 2011; Griese *et al.* 2011; Lange *et al.* 2011; Spaans *et al.* 2015; Zerulla *et al.* 2016), emphasizing the reliablity of this method. With the aim of accuracy, two loci (TTC1610 (near *oriC*) and TTC0574 (relatively closer to *terC*) on the chromosome was chosen as the investigation targets. The copy numbers of the two loci determined by the real-time PCR method are summarized in Table 3, which clearly showed that multiple sets of chromosomes existed per cell during the exponential growth phase in *T*. *thermophilus* HB27, and this is consistent with the study performed in the strain HB8 (Ohtani *et al.* 2010).

**Table 3:** Chromosome copy numbers in *T. thermophilus* HB27

Gene locus	Real-time PCR*^a^*	Fluorescent microscopy*^b^*
TTC1610 (*oriC* region)	7.87 ± 0.73	7.53 ± 2.71
TTC0574 (relatively closer to *terC*)	7.01 ± 1.55	/

a real-time PCR method was used to measure the chromosome copy numbers.

b Fluorescent microscopy was used to count ParB-sGFP fluorescent foci numbers per cell reflecting the copy numbers of the chromosomal origin region (100 cells were counted).

### Heterozygous T. thermophilus HB27 cells and their **g**enetic outcomes

**Figure 1:**
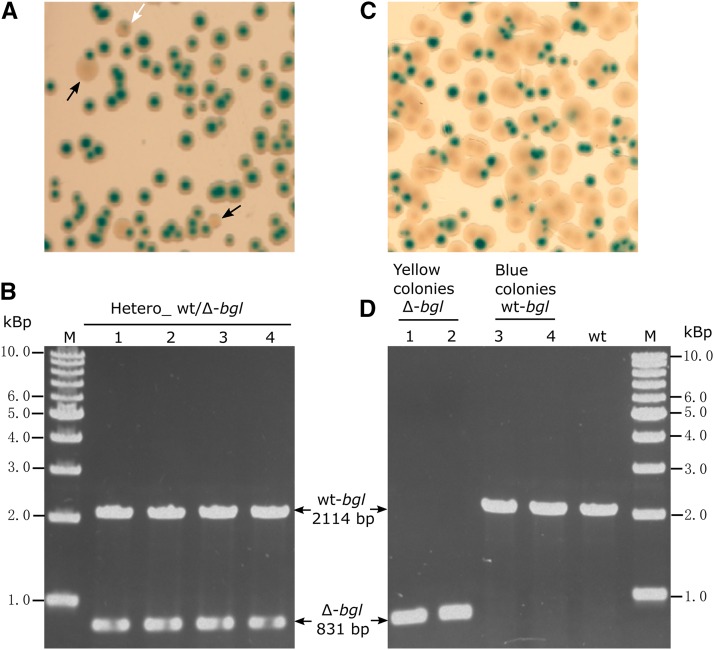
Heterozygous wt/Δ-*bgl* cells and their genetic outcomes. (A) Phenotype of homozygous and heterozygous Δ*bgl* transformants on TB-X-Gluc plate. Black arrows: yellow colonies (homozygous Δ*bgl*); white arrow: colony with intermediate color (heterozygous wt/Δ-*bgl*). (B) Genotypes of the transformants showing intermediate phenotype were determined by PCR (using primers binding the flanking regions of *bgl*). (C, D) The genetic outcomes of the heterozygous wt/Δ-*bgl* strain. The phenotype (C) and genotype (D) of the progeny of the heterozygous strain after growing without selection and re-streaking on TB-X-Gluc plate. wt-*bgl:* genotype of the blue colonies from (C), Δ-*bgl*: genotype of the yellow colonies from (C).

Since *T*. *thermophilus* HB27 is polyploid, it is conceivable that under certain condition, heterozygous cells containing two different alleles at one gene locus would appear. In practice, this kind of heterozygous cells was previously noticed during studies of *T. thermophilus* HB27 when generating deletion mutants of non-essential genes. Take *bgl* gene for example, wild-type *T. thermophilus* HB27 cells express β-glucosidase (Bgl). It is easy to distinguish Bgl^-^ colonies (yellow) from Bgl^+^ colonies (blue) on agar plates supplemented with indicator substrate, *i.e.*, X-Gluc (Ohta *et al.* 2006). Interestingly, when creating Δ*bgl* mutant through direct double-crossover homologous recombination method, although apparent homozygous Δ*bgl* mutant (yellow color) could be easily detected on TB X-Gluc plates (Figure 1A), transformants which projected intermediate phenotype (the color was between yellow and blue) could also be found (Figure 1A). PCR analysis showed that these “intermediate” colonies were carrying both knockout and wild-type alleles simultaneously at the *bgl* locus (Figure 1B). However, they were genetically unstable, since after growing in liquid medium without selection followed by re-streaking on agar plates with X-Gluc, colonies showing homozygous for the knockout or wide-type *bgl* allele were observed (Figure 1C, D). This result indicated that *T*. *thermophilus* cells could form heterozygous state carrying different alleles at one locus, and without selection pressure, the alleles could segregate resulting in different genetic outcomes in the progeny.

### Construction of a stable heterozygous strain

**Figure 2:**
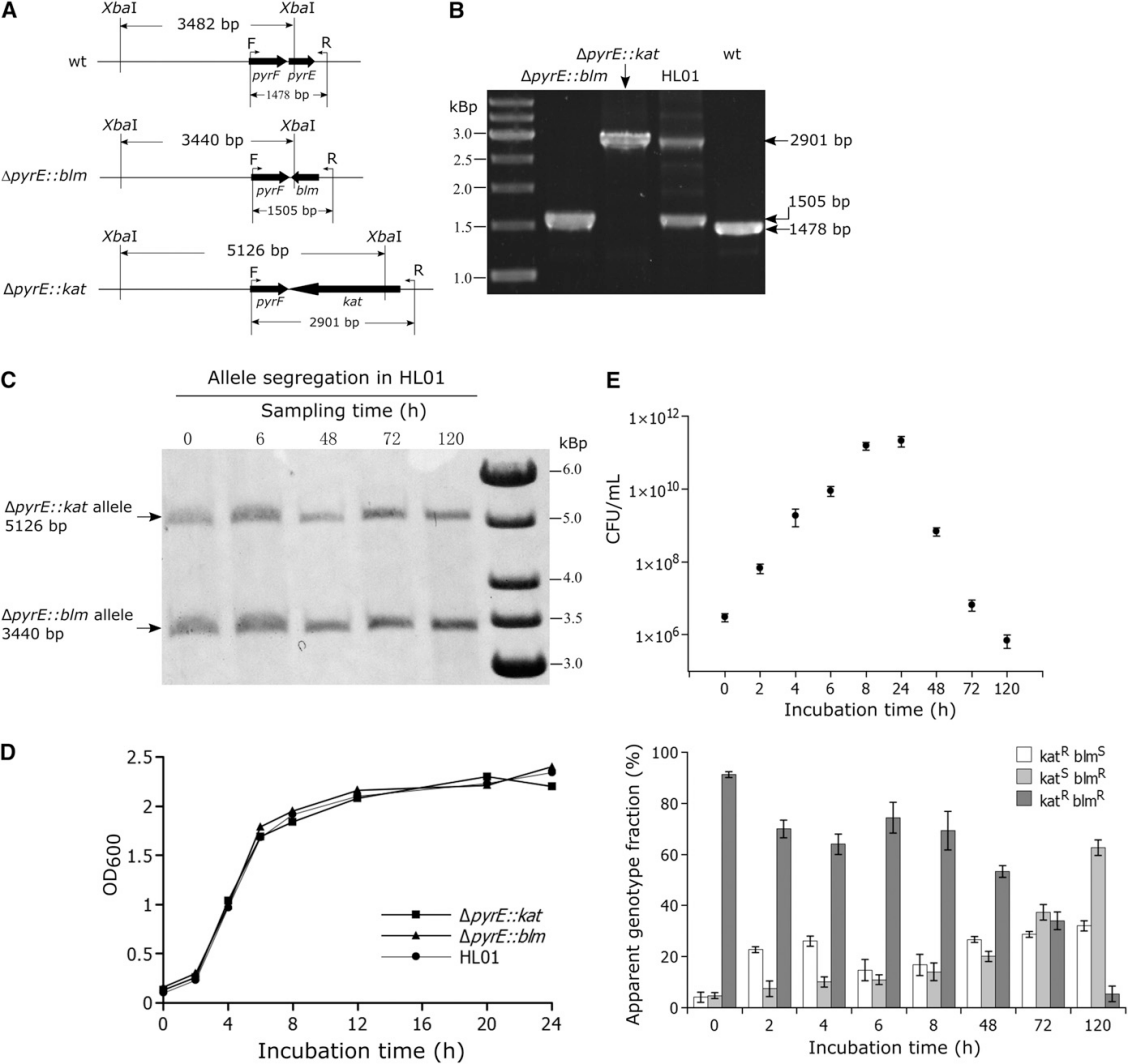
Genotype confirmation and allele segregation kinetics of the HL01 strain. (A) Schematic drawings of the genotypes of the Δ*pyrE*::*blm and* Δ*pyrE*::*kat* strains. *Xba*I site was found in *pyrE*, *blm* and *kat* respectively. (B) PCR confirmation of the Δ*pyrE*::*blm*, Δ*pyrE*::*kat* and HL01 strains using primers F and R (the positions are shown in A). The predicted sizes of the PCR products were 1478 bp for the wild type, 2901 bp for Δ*pyrE*::*kat*, 1505 bp for Δ*pyrE*::*blm*, and both 2901 bp and 1505 bp for HL01. (C, E) Apparent genotype and allele abundance measurements of the heterozygous strain HL01 grown in liquid TB medium at 70° in the absence of selection. (C) The changes in the relative abundance of the two alleles (*kat* and *blm*) were acquired by measuring the intensity of the bands from the Southern blot. For the Southern blot, a 450 bp biotin-labeled fragment of the *pyrF* gene was used as the probe for hybridization; genomic DNA was prepared from samples taken at the indicated time points and was digested with *Xba*I. The *in silico* predicted sizes are 5126 bp and 3440 bp for the Δ*pyrE*::*kat* and Δ*pyrE*::*blm* alleles, respectively. One representative of three independent analyses is shown. (E) The viable counts were determined from the antibiotic-free plates (upper panel). The changes in the fraction of each phenotype (Kat^R^Blm^R^ – dark-gray bar, Kat^R^Blm^S^ – white bar and Kat^S^Blm^R^ – light-gray bar) in the population were followed by spreading the samples on antibiotic-free plates and restreaking 50 colonies for each time point on plates containing kanamycin or bleomycin (lower panel). Shown are mean and standard deviation from the three independent experiments. (D) The cell growth curves of the Δ*pyrE*::*blm*, Δ*pyrE*::*kat* and HL01 strains.

In order to investigate what mechanism had drived the allele segregation phenomenon, a stable heterozygous strain was constructed by inserting two different selection markers at the same chromosomal locus (*pyrE*). To this end, initially, two homozygous strains (Δ*pyrE*::*kat* and Δ*pyrE*::*blm*) were created by replacing the *pyrE* gene with a kanamycin and a bleomycin resistant cassette, respectively (Figure 2A). Numerous examples have shown that homozygous gene deletion mutant could be obtained in *T. thermophilus* in spite of its polyploid genomic background (Angelov *et al.* 2013; Leis *et al.* 2014; Li *et al.* 2015; Ohtani *et al.* 2015; Wang *et al.* 2016). In the experience, when a non-essential gene was targeted by a vector containing an antibiotic resistant marker sandwiched by two homology arms of that target region, near 90% of the resultant transformants were homozygous deletion mutant of that gene. In the generation of Δ*pyrE*::*kat* and Δ*pyrE*::*blm*, eight transformants were respectively chosen for PCR determination of the deletion mutants, and only one transformant was found to contain the wild-type *pyrE* allele (Figure S1). The possibility of obtaining homozygous gene deletion mutants with high frequency in the polyploid *T. thermophilus* genome, again suggested that after the mutant allele has been introduced into the specific genomic region, allele segregation would lead to homozygous mutant cells. Thereafter, the homozygous Δ*pyrE*::*kat* strain was transformed with linearized pJ-Δ*pyrE*::*blm* plasmid. Selection on TB agar plates supplemented with both antibiotics allowed to generate the heterozygous strain HL01. PCR, Southern blot and DNA sequencing was used to verify the genotype of the heterozygous strain, which excluded the possibility of vector integration and unequivocally showed that in HL01 the *pyrE* gene was completely replaced by both the *kat* and *blm* resistance cassettes and no wild-type *pyrE* allele was detectable (Figure 2A, B, C). The heterozygous state of the HL01strain could be stably maintained in medium supplemented with both kanamycin and bleomycin. The cell growth curves showed that there were no growth rate difference among the two homozygous strains (Δ*pyrE*::*kat* and Δ*pyrE*::*blm*) and the heterozygous strain HL01 (Figure 2D).

### Allele segregation kinetics in the heterozygous strain HL01 in the absence of selection

To detect and quantify allele segregation in the heterozygous strain, HL01 was continuously grown in the absence of selection pressure for 120 h (without dilution or regrowth from stationary phase). Samples were withdrawn at different time points and were used to determine: i) the drug resistance phenotype of 50 individual colonies per time point and ii) the relative frequency of the two alleles in the samples, using Southern blot followed by band intensity measurement. The drug resistance profile implies the genotype of the tested colony (homozygous for either the *kat* or the *blm* allele, or heterozygous), while changes in the relative abundance of each allele in the whole population could be detected by Southern blot. In this experiment, it was observed that during growth in the absence of selection pressure the fraction of heterozygous cells (as determined by the resistance phenotype) decreased and that of homozygous cells increased (Figure 2E). The Southern blot analysis showed that, despite the significant changes in the observed genotypes, the relative amount of *kat*/*blm* allele in the whole population kept almost constant throughout the experiment (Figure 2C). Apart from random chromosome partitioning, another mechanism, which is gene conversion (Pecoraro *et al.* 2011; Lange *et al.* 2011), could serve to explain the allele segregation phenomenon as well. However, in addition to changing the genotype frequency, gene conversion also leads to changes in the allele abundance in the whole population (see discussion). Since the relative amount of *blm*/*kat* allele did not change, gene conversion was ruled out. Taken together, the data from this experiment indicated that random chromosome segregation is the most likely mechanism leading to allele segregation in the heterozygous cells when they were grown in the absence of antibiotic selection pressure.

### Distribution of DNA content in two dividing daughter cells

**Figure 3:**
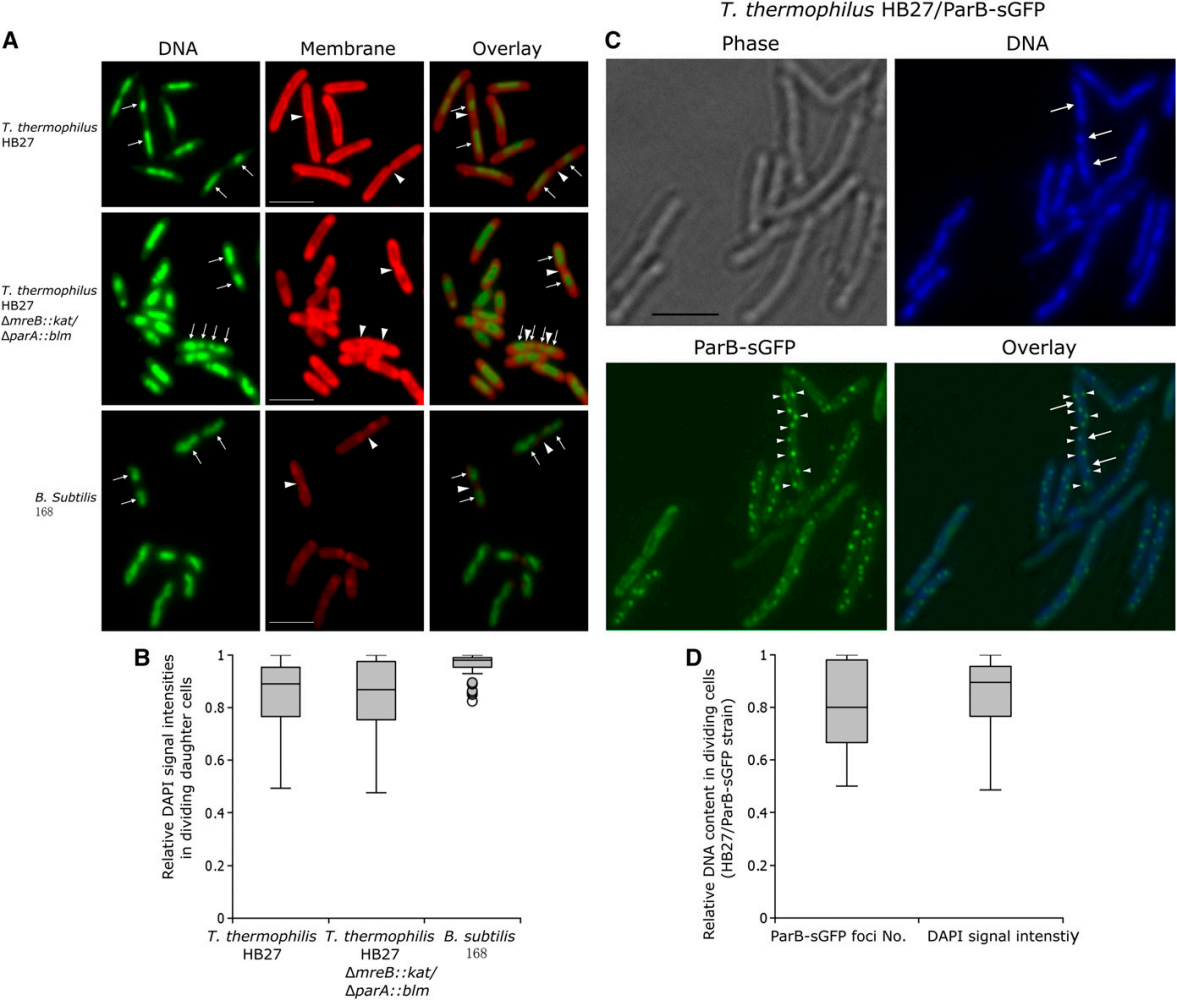
Relative DNA contents in dividing cells of *T. thermophilus* HB27, HB27/ParB-sGFP, Δ*mreB*::*kat/*Δ*parA*::*blm*, and *B. subtlis* 168. (A) Representative images of exponentially growing and dividing *T. thermophilus* and *B. subtlis* cells stained with 6-carboxyfluorescein (membrane) and DAPI (DNA) used in the analysis of the DNA intensity scatter. The white arrows show examples of separated nucleoids in cells where the formations of septa are visible (triangles), bars indicate 2 µm. (B) Box plot of the distribution of DAPI intensity in 100 randomly selected dividing *T. thermophilus* HB27, Δ*mreB*::*kat/*Δ*parA*::*blm* and *B. subtilis* cells. The fluorescence intensity ratio in the two daughter cells was calculated by dividing the intensity value of the cell with lower fluorescence signal by the one with a higher one. The whiskers represent the minimum and the maximum of all the data, circles show outliers. (C) The *T. thermophilus* chromosomal *parAB* – *parS* system was used to determine relative DNA contents (*i.e.*, relative numbers of the *oriC* region) in dividing cells. Representative images show cells with different numbers of ParB-sGFP/*parS* fluorescent foci in the two daughter nucleoids (white triangles: fluorescent foci, white arrows: sister nucleoids, bar indicates 2 µm). (D) Box plot of the distributions of relative ParB-sGFP/*parS* fluorescent foci numbers and relative DAPI signal intensities in 100 dividing HB27/parB-sGFP cells. The ratio was achieved by dividing the lower value by the higher one.

In bacterial *parAB* – *parS* systems, ParB can bind its *cis*-acting element *parS* (Lin and Grossman 1998). In *T. thermophilus*, it has been previously shown that the chromosomal *parS* site is located near the chromosomal *oriC*, and that ParB-sGFP fusion proteins could bind *parS* forming fluorescent foci (Li *et al.* 2015). The fluorescent foci numbers per cell to some extent represent the copy numbers of *parS*, *i.e.*, of the chromosomal *oriC* region. Based on these observations, and to further confirm that the chromosome partitioning mode in *T. thermophilus* is non-stingent and often variable, the DNA content in dividing daughter cells was measured based on the signal intensities of DAPI-stained nucleoids, as well as on the numbers of fluorescent foci formed by ParB-sGFP/*parS* protein-DNA complexes. The two methods have been used also in studying of random chromosome segregation pattern in cyanobacterial species (Schneider *et al.* 2007; Hu *et al.* 2007). Specifically, exponentially growing cells of *T. thermophilus* HB27 were stained with DAPI (a DNA stain) and 6-carboxyfluorescein (CFS, a membrane stain), and the relative DNA staining intensities of 100 dividing cells (cells in which the nucleoids were separated and the division septum was visible) were measured. *B. subtilis* 168 was stained and analyzed in the same way and was used as a control in which stringent chromosome partitioning has been shown. Representative images from these measurements are shown in Figure 3A. The fluorescence signals of the two daughter nucleoids in dividing *T. thermophilus* cells (Figure 3A, top row; Figure S2) revealed unequal intensities of the nucleoids in contrast to *B. subtilis* cells, in which an almost equal distribution of the DAPI signal in the two daughter cells was observed (Figure 3A, bottom row). Further, it seems that in the *T. thermophilus* dividing cells with unequal amount of sister nucleoids, septa were also not always formed exactly at mid-cell positions (Figure 3A, top row; Figure S2). Figure 3B shows the relative DNA content, determined by DAPI signal intensity, in 100 pairs of daughter nucleoids in dividing cells of *T. thermophilus* and in *B. subtilis*. In the *B. subtilis* cells, the signal ratios distributed from 0.82 to one (mean 0.96, SD 0.04), while in the *T. thermophilus* cells, the relative ratios had a much broader distribution (from 0.49 to one with a mean of 0.84 and a SD of 0.15). In the polyploid *Synechocystis* sp., a similar analysis has been performed (Schneider *et al.* 2007), in which the relative DNA contents in the *B. subtilis* dividing cells varied from 0.76 to one, with a mean value of 0.94 and a SD of 0.05, emphasizing the reproducibility of this method. The above analysis suggested that unequal distribution of the genetic material occurred in the dividing *T. thermophilus* cells. It seems that although chromosome could segregate irregularly, the frequencies of anucleate cells were actually low, only 1.25% cells were found to be anucleate based on the fluorescent microscopic analysis. The phenomenon was also observed in cyanobacterium *Anabaena* sp. PCC 7120 which has been suggested to exert random chromosome segregation as well (Hu *et al.* 2007). This may be due to the fact that the daughter cells of these polyploid bacteria are likely to get at least one chromosome by chance, and subsequent chromosome replication may compensate for variance created during cell division.

The above results were further verified by using the *T. thermophilus parAB* – *parS* system. For this, a C-terminal sGFP fusion of the chromosomally encoded ParB protein was constructed (Li *et al.* 2015), and the ParB-sGFP fusion protein was expressed from the replicative plasmid pMK18 (de Grado *et al.* 1999). When expressed in *T. thermophilus* HB27, well-defined fluorescent foci could be observed (Figure 3C). The fluorescent foci numbers represent the copy numbers of *parS* sites, as has been shown previously (Li *et al.* 2015). In the example image Figure 3C, five to 12 fluorescent foci could be observed in the growing cells with dividing nucleoids, indicating five to 12 copies of *parS* (and of *oriC* region) existed in one cell, again confirming the polyploidy of *T. thermophilus* (Table 3). The pairwise distribution of fluorescent foci numbers in 100 pairs of daughter cells was almost identical to the distribution of the relative DAPI signals (Figure 3D), with a mean of 0.83 and a SD of 0.16. This result further confirmed that random chromosome segregation could occur in the polyploid *T. thermophilus* cells.

### Inactivation of parA and mreB in T. thermophilus HB27

**Figure 4:**
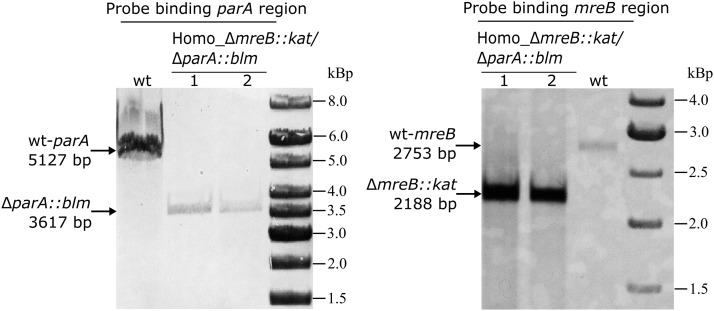
Generation of the Δ*mreB*::*kat/*Δ*parA*::*blm* double deletion mutant in *T. thermophilus*. The *parA* and *mreB* genes were replaced by *blm* and *kat* cassette respectively, genotype confirmation of Δ*mreB*::*kat/*Δ*parA*::*blm* was performed by Southern blot, using probes binding the *mreB* and *parA* regions for hybridization respectively. The genomic DNAs were digested with *Bam*HI for confirmation of *parA* deletion, and were digested with *Bgl*II for confirmation of *mreB* deletion. The *in silico* predicted sizes are respectively indicated with arrows on the left side of each image.

The Par system and actin-like protein MreB have been shown to participate in active chromosome partitioning in many bacterial species. By searching out the complete genome of *T. thermophilus* HB27 (Henne *et al.* 2004), only one copy of chromosomally encoded ParA and MreB homolog could be identified. And given their important role in other bacteria, their function with respect to chromosome segregation was investigated in this species. The *mreB* and *parA* genes were deleted by replacing them with the *kat* and *blm* resistance markers respectively (a schematic design see Figure S3A). Initially, homozygous Δ*mreB*::*kat* was generated (Figure S3B). The suicide vector pUC-Δ*parA*::*blm* was then transformed into the correct Δ*mreB*::*kat* strain, and selected on agar plates with both antibiotics (see materials and methods). Seven colonies were randomly selected and exposed to PCR prescreen, this was to determine transformants which contained homozygous Δ*parA*::*blm* allele in the Δ*mreB*::*kat* background. Six colonies were found to be homozygous Δ*mreB*::*kat*/Δ*parA*::*blm* mutants (Figure S3C). Southern blot analysis further confirmed that the double deletion mutant had correct insertion of *kat* and *blm* at the *mreB* and *parA* loci respectively, and no wild-type alleles were left (Figure 4). To investigate potential chromosome segregation defects in the mutant, exponentially growing cells were collected, stained by DAPI and CFS and analyzed by fluorescence microscopy. In dividing Δ*mreB*::*kat*/Δ*parA*::*blm* cells, the replicated nucleoid could segregate normally to the daughter cells (a representative image is shown in Figure 3A, middle row). Furthermore, compared with the wild type, no considerable increase in the frequency of anucleate or DNA-less cells in Δ*mreB*::*kat*/Δ*parA*::*blm* was observed (1.25% in the wild type and 1.50% in the mutant, 400 cells were analyzed), suggesting that the deletion of *parA* and *mreB* in *T. thermophilus* has no effect on the chromosomal bulk nucleoid segregation. The relative DNA content in 100 pairs of dividing cells, measured by DAPI signal intensities, were plotted as well. The relative DNA content in the dividing daughter cells of Δ*mreB*::*kat*/Δ*parA*::*blm* distributed from 0.48 to one, with a mean of 0.85 and a SD of 0.13. This data are almost indistinguishable from that of the wild type strain (Figure 3B). Taken together, the results suggested that in *T. thermophilus*, ParA and MreB do not actively participate in the chromosomal bulk DNA segregation process. A similar observation has been reported for the cyanobacterium *Anabaena* sp. PCC 7120, which is also polyploid (Hu *et al.* 2007), whereas in haploid bacteria such as *B. subtilis* and *C. cresentus*, ParA and MreB have been shown to be involved in chromosomal bulk DNA and origin segregations (Ireton *et al.* 1994; Lewis and Errington 1997; Mohl and Gober 1997; Soufo and Graumann 2003; Gitai *et al.* 2004, 2005; Shebelut *et al.* 2009, 2010; Scholefield *et al.* 2011).

## Discussion

### Allele segregation in heterozygous T. thermophilus cells

This work showed that the *T. thermophilus* strain HB27 contains on average seven to eight chromosomal copies per cell when grown in rich medium. This number was estimated using two independent methods, *e.g.*, a real time PCR-based method and a fluorescence microscopy method, based on counting the numbers of fluorescent ParB-sGFP/*parS* foci per cell. The estimation of the chromosomal copy number is in good agreement with the one reported in another *T. thermophilus* strain, HB8 (Ohtani *et al.* 2010). One of several possible explanations for polyploidy is that this strategy favors the survival of bacteria living under harsh conditions. For example, due to multiple genome copies, *D. radiodurans* is able to rapidly reassemble the chromosomes destroyed by desiccation and ionizing radiation (Zahradka *et al.* 2006; Slade *et al.* 2009). *T*. *thermophilus* which grows at temperatures up to 85° may also suffer frequent DNA damage, and polyploidy may facilitate DNA repair by homologous recombination.

An interesting phenomenon observed repeatedly in *T. thermophilus* is the allele segregation in heterozygous cells containing two different alleles in one locus (Figure 1). It was considered that this could be the result of either random chromosome segregation or gene conversion. While both processes can result in homogeneity in the progeny, their intrinsic mechanisms are different. Gene conversion is essentially a non-reciprocal exchange of information between homologous chromosomes as well as between repeated sequences on the same chromosome (Paulsson *et al.* 2017). In principle, for a heterozygous cell containing both mutant and wild-type allele at one locus, gene conversion can conditionally occur in both directions, *e.g.*, if the mutant allele is converted against the wild-type allele, the abundance (copies) of the mutant allele will be eliminated and that of the wild-type allele will be increased, and the reverse is also true (Khakhlova and Bock 2006; Soppa 2011). In polyploid archaea, allele equalization among the multiple genome copies via gene conversion has been repeatedly demonstrated (Hildenbrand *et al.* 2011; Lange *et al.* 2011; Guschinskaya *et al.* 2016). For instance, Lange *et al.* (2011) showed that in *Haloferax volcanii*, the heterozygous strain *leuB*/Δ*leuB*::*trpA* would convert to homozygous *leuB* or Δ*leuB*::*trpA* strain. In the presence of tryptophan, Δ*leuB*::*trpA* allele was converted to *leuB* allele, resulting in the increase of the fraction of homozygous *leuB* strain, in the meanwhile, the abundance of the Δ*leuB*::*trpA* allele was eliminated and that of the *leuB* allele was accordingly increased in the whole population; in the presence of leucine, the conversion was occurred in the opposite direction. On the other hand, if random chromosome segregation occurred in polyploid cells carrying two different alleles at one gene locus, daughter cells tended to receive different combination and/or numbers of the replicated parental chromosomes, which would also lead to change in the genotype frequency of the progeny, however, the relative allele abundance would be always maintained. To be able to discriminate which mechanism had led to the observed allele segregation in the heterozygous *T. thermophilus* cells, a stable heterozygous strain (HL01) containing two different markers (*kat* and *blm*) at the *pyrE* locus was generated (Figure 2B, C). When this strain was grown in the absence of antibiotic selection, a gradual segregation of the two markers was observed (Figure 2E). In this experiment the fraction of homozygous cells increased significantly, while at the same time the relative abundance of *kat* and *blm* in the population remained constant (Figure 2C, E). This indicates that the allele segregation was probably triggered by chromosome random segregation but not by gene conversion (see above). Interestingly, during the allele segregation kinetics, the fraction of the heterozygous strain decreased more steady in the stationary phase than in the exponential phase (*e.g.*, the fraction was increased at the time point 6 h and 8h compared with 4 h). This may be explained by the high frequency of natural transformation in the exponential growth phase of *T. thermophilus* HB27. Although cell divisions proceeded faster in the exponential phase, DNA exchange among the cells (due to the uptake by natural transformation of free DNA released by dead cells) might also occur (César *et al.* 2011; Alvarez *et al.* 2011). Such an exchange would counteract allele segregation and would affect the frequency of the apparent genotypes of the two alleles. Indeed, addition of EDTA, which is known to impede DNA uptake, to the allele segregation reactions resulted in a more rapid segregation of the two alleles in the exponential phase (2 h - 8 h) (Figure S4). Another mechanism contributing to the steady allele segregation in the late stationary phase may be reductive cell division (Kolter *et al.* 1993; Navarro Llorens *et al.* 2010). Such reductive cell division has been shown to cause decrease in the average genome copy numbers in many polyploid bacteria and archaea, as shown by the extreme example, the copy numbers of cyanobacterium *Synechocystis* PCC 6803 could decrease from 218 copies in exponential growth phase to 58 copies in stationary growth phase (Griese *et al.* 2011). In *T. thermophilus*, the average chromosome copy number per cell was four to five under slow growth condition (Ohtani *et al.* 2010), while that was seven to eight in fast-growing culture, indicating that the value is also conditionally regulated. Therefore, it is conceivable that in this experiment, the decrease of average chromosome copies in the stationarily-growing cells would actually favor the daughter cells receive the same type of parental chromosome by random partitioning.

### Chromosome random partitioning in polyploid bacterial cells

The complete segregation of bacterial chromosomes undergoes three separated steps: separation of the replicated origin regions, segregation of the replicated bulk chromosome, and finally resolution of the termini at the division septum (Wang and Rudner 2014; Badrinarayanan *et al.* 2015). Nevertheless, the molecular mechanisms involved in the chromosome segregation in non-model bacteria are only just beginning to be elucidated. Most sequenced bacterial genomes (with the exception of *E. coli*) contain a chromosomally encoded *par* locus, which is consisted of three components: two *trans*-acting proteins (ParA and ParB) and a *cis*-acting site (*parS*). The ParAB – *parS* system is believed to drive active chromosome segregation in many bacteria, including origin and/or bulk DNA segregations. Deletion of *par* genes is lethal in some bacteria, *e.g.*, in *C. crescentus* (Mohl and Gober 1997; Mohl *et al.* 2001), *V. cholera* (*parAB2*) (Yamaichi *et al.* 2007) and *Myxococcus xanthus* (Iniesta 2014). In *C. crescentus*, the ParA retraction has been shown to mediate the latter part of ParB translocation, thus is crucial for the final stages of chromosome segregation (Shebelut *et al.* 2010). Deletion of the *parAB2* genes located on chromosome II of *V. cholera* could lead to localization and segregation defects of the chromosome II, and yield cells with only chromosome I (Yamaichi *et al.* 2007). In some other species, *par* mutants are viable but exhibit various severity defects of chromosome bulk DNA or origin segregation in the vegetative and/or sporulation phase of growth, *e.g.*, in *B. subtilis*, *S. coelicolor*, *V. cholera* (*parAB1* of chromosome I), *P. aeruginosa*, *C. glutamicum*, *S. pneumoniae*, and *M. smegmatis* (Ireton *et al.* 1994; Sharpe and Errington 1996; Lewis and Errington 1997; Kim *et al.* 2000; Jakimowicz *et al.* 2002; Fogel and Waldor 2006; Lasocki *et al.* 2007; Murray and Errington 2008; Sullivan *et al.* 2009; Gruber and Errington 2009; Donovan *et al.* 2010; Scholefield *et al.* 2011; Minnen *et al.* 2011; Ginda *et al.* 2013). The bacterial actin-like protein MreB has also been suggested to be involved in chromosome segregation in addition to its role in cell morphology determination (Sundararajan and Goley 2017). In *B. subtilis*, Soufo and Graumann (2003) showed that depletion of *mreB* led to the appearance of 25% anucleate cells, and depletion of the other two *mreB* homologs (*mbl* or *mreBH*) also caused a considerable increase in the fraction of anucleate cells. In *E. coli*, the initial studies truly showed that MreB is necessary for origin and bulk nucleoid segregation; when the *E. coli* cells were treated with A22 which is a new antibiotic specifically targeting MreB, chromosome segregation was inhibited, resulting in cells containing large confluent bodies of nucleoids (Kruse *et al.* 2003, 2006). In *C. cresentus*, alterations in MreB expression also cause defects in chromosome segregation (Gitai *et al.* 2004, 2005). The A22 treated *C. cresentus* cells experienced growth condition-specific defects in segregation of the chromosomal origin-proximal regions (Gitai *et al.* 2005; Shebelut *et al.* 2009). In this work, the *T. thermophilus parA* and *mreB* gene were simultaneously deleted by replacement with antibiotic-resistant genes. The results showed that the *parA* and *mreB* deletion mutant had a similar chromosome partitioning pattern as the wild type (Figure 3A, B), indicating no apparent chromosome segregation defects occurred in the Δ*mreB*Δ*parA* muant. Thus, both the Par and MreB systems are not involved in the chromosomal bulk DNA segregation in *T. thermophilus*. A similar result was found in the polyploid cyanobacterium *Anabaena* sp. PCC 7120, in which the lack of *mreB* did not lead to any DNA segregation defects in the two dividing daughter cells (Hu *et al.* 2007). Still, it is of interest what the function is of the *parABS* and *mreB* loci in *T. thermophilus* when they are not critical for cell growth and chromosomal DNA segregation. The *parABS* loci are located extremely approching *oriC* (appoximately 6 kb distance) (Li *et al.* 2015), and ParB could specifically bind *parS* forming nucleoprotein complexes spreading around the origin region, indicating that *parABS* are actually functional. Thus, *parABS* may contribute to other cell functions, such as serve to be transcriptional regulators. Recent studies have shown that analogous to their counterparts on the plasmids, the chromosomal Par proteins can also regulate transcriptions of other genes located around *parS* sites (Bartosik *et al.* 2014; Baek *et al.* 2014; Attaiech *et al.* 2015). For example, in *P. aeruginos*a, deletion of *parB* leads to global transcriptional changes affecting more than 1000 genes (Bartosik *et al.* 2014). Thus, it will prove interesting to test whether this is the situation for the *T. thermophilus* Par system in the future. Further, although MreB did not participate in the chromosome segregation, it was found to be a cell shape determinant in *T. thermophilus*. The cells of the Δ*mreB*::*kat*/Δ*parA*::*blm* double gene deletion mutant and Δ*mreB*::*kat* single gene deletion mutant have become much thicker and rounder compared with those of the wild-type strain (Figure 3A, middle row). The average ratio of cell length/diameter was 4.24 ± 0.21 in Δ*mreB*::*kat*, while that in the wild type was 8.71 ± 0.86, in addition, a certain number of the mutant cells have become drastically abnormal (not shown).

Taken together, it seems that the specific protein-based chromosome segregation machineries, *e.g.*, ParAB – *parS* system and/or MreB are critical for certain bacteria containing a single chromosome during most of the cell cycles, as they ensure that the daughter cells receive an equal number of the parental chromosome during separation of sister chromosomes. Alternatively, bacteria normally containing multiple chromosomes per cell may not require an active segregation mechanism, since the frequency of daughter cells receiving none of the parental chromosomes is actually low (Graumann 2010). This is analogous to high-copy-number plasmid systems, which typically lack an active segregation mechanism (Ebersbach and Gerdes 2005).

An intriguing question is how the polyploid bacteria segregate their chromosome copies to the daughter cells. The allele segregation kinetics experiment in the heterozygous *T. thermophilus* strain HL01 suggested that the chromosome segregation mode in *T. thermophilus* is variable, and random segregation could occur. This suggestion was strengthened by monitoring the relative DNA content in dividing cells via DAPI-staining and by *in vivo* monitoring of ParB-sGFP/*parS* protein-DNA complexes (Figure 3). Similar conclusions were drawn for the polyploid cyanobacteria *Anabaena* sp. PCC 7120 (Hu *et al.* 2007) and *Synechocystis* sp. (Schneider *et al.* 2007), and the polyploid euryarchaeota *M. jannaschii* (Malandrin *et al.* 1999). In these prokaryotes, it would be possible that nonprotein-based chromosome segregation mechanisms, *e.g.*, physical forces from extrusion of DNA from replication forks may help push it toward opposite poles (Jun and Mulder 2006; Jun and Wright 2010; Fisher *et al.* 2013; Badrinarayanan *et al.* 2015), thereby random chromosome segregation occur frequently. The observation that septa were not always formed exactly at the middle position of *T. thermophilus* cells could actually support the proposal. Further, it is worthy to note that the relaxation of DNA segregation control may not be a general trait for all polyploid bacteria or archaea. It has been shown that chromosomes were segregated precisely in the polyploid archaeon, *Halobacterium salinarum* (Breuert *et al.* 2006) as well as in the polyploid cyanobacterium, *Synechococcus elongates* (Jain *et al.* 2012).
